# Petosemtamab, a Bispecific Antibody Targeting Epidermal Growth Factor Receptor (EGFR) and Leucine-Rich G Repeat-Containing Protein-Coupled Receptor (LGR5) Designed for Broad Clinical Applications

**DOI:** 10.3390/cancers17101665

**Published:** 2025-05-14

**Authors:** Ante S. Lundberg, Cecile A. W. Geuijen, Sally Hill, Jeroen J. Lammerts van Bueren, Arianna Fumagalli, John de Kruif, Peter B. Silverman, Josep Tabernero

**Affiliations:** 1 Merus NV, 3584 CT Utrecht, The Netherlands; 2 Vall d’Hebron Institute of Oncology (VHIO), Universitat de Vic/Central de Catalunya (UVic-UCC), 08035 Barcelona, Spain

**Keywords:** petosemtamab, bispecific antibody, head and neck cancer, colorectal cancer, cancer biology, cancer stem cell, LGR5 protein, EGFR protein

## Abstract

Disease progression and treatment resistance in colorectal and other cancers are driven by a subset of cells within the tumor that have stem-cell-like properties and long-term tumorigenic potential. These stem-cell-like cells express the leucine-rich G repeat-containing protein-coupled receptor 5 (LGR5) and have characteristics similar to tissue-resident stem cells in normal adult tissues such as the colon. Organoid models of murine and human colorectal and other cancers contain LGR5-expressing (LGR5+) stem-cell-like cells and can be used to investigate the underlying mechanisms of cancer development, progression, therapy vulnerability, and resistance. A large biobank of organoids derived from colorectal cancer or adjacent normal tissue was developed. We performed a large-scale unbiased functional screen to identify bispecific antibodies (BsAbs) that preferentially inhibit the growth of colon tumor-derived, as compared to normal tissue-derived, organoids. We identified the most potent BsAb in the screen as petosemtamab, a Biclonics^®^ BsAb targeting both LGR5 and the epidermal growth factor receptor (EGFR). Petosemtamab employs three distinct mechanisms of action: EGFR ligand blocking, EGFR receptor internalization and degradation in LGR5+ cells, and Fc-mediated activation of the innate immune system by antibody-dependent cellular phagocytosis (ADCP) and enhanced antibody-dependent cellular cytotoxicity (ADCC) (see graphical abstract). Petosemtamab has demonstrated substantial clinical activity in recurrent/metastatic head and neck squamous cell carcinoma (r/m HNSCC). The safety profile is generally favorable, with low rates of skin and gastrointestinal toxicity. Phase 3 trials are ongoing in both first-line programmed death-ligand 1-positive (PD-L1+) and second/third-line r/m HNSCC.

## Simple Summary

Tumors of the aerodigestive tract such as colorectal and head and neck cancers contain specific cells that contribute to disease progression and resistance to treatment. These cancer driver cells express the leucine-rich G repeat-containing protein-coupled receptor 5 (LGR5) at the cell surface. Petosemtamab is a bispecific antibody that binds to both LGR5 and epidermal growth factor receptor (EGFR). EGFR is a cell surface receptor involved in controlling cell division and cell survival. This review outlines the role of LGR5 in the growth and metastasis of colorectal and other cancers, as well as the discovery and development of petosemtamab. Petosemtamab was selected from approximately 500 different bispecific antibodies tested for their ability to preferentially inhibit the growth of organoids derived from colon cancer but not those derived from normal tissue. This review also discusses the unique mechanisms of action of petosemtamab as well as promising early clinical results in the treatment of head and neck cancer.

## 1. Introduction

**Figure 1:**
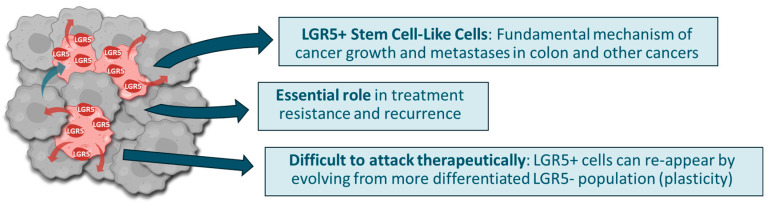
Epithelial malignancies are heterogeneous collections of cells. A small fraction of these cells express LGR5 and share other characteristics of stem cells. These stem-cell-like cells have an essential role in driving cancer growth and metastases, as well as resistance to treatment and recurrence. LGR5+ cells are difficult to attack therapeutically.

Advanced and metastatic cancers are generally considered incurable. Achieving complete and long-lasting disease eradication with current therapies remains a significant challenge. Most patients inevitably experience recurrence or disease progression. Epithelial cancers are typically heterogeneous masses of cells, in which only a minor subset of cells appears to be responsible for cancer growth, metastases, and treatment resistance [1,2,3,4,5,6,7]. These more malignant “cancer driver” cells have been described as less differentiated, more stem-cell-like, and displaying partial epithelial to mesenchymal transition [8,9]; they are also thought to be a major source of treatment failure [10]. In colorectal cancer (CRC), for example, the subset of malignant cells that drive cancer growth has been well described (Figure 1). They appear to be similar to the non-malignant resident adult intestinal stem cells (ISCs), having characteristics of self-renewal, long-term replicative potential, and multipotency, that is, the capacity to give rise to short-lived terminally differentiated cells [3,4,7,11]. In addition, both ISCs and CRC stem-cell-like cells are characterized by the expression of the Wingless-related integration site (WNT) signaling pathway protein LGR5 [7,11].

One important characteristic of ISCs is the capacity for phenotypic plasticity. For example, damage to the colonic epithelium can lead to the loss of the population of endogenous LGR5+ ISCs. In this setting, LGR5- cells within the colonic crypt have been shown to display phenotypic plasticity and differentiate into LGR5+ cells. Notably, this capacity for phenotypic plasticity is also found in cells within human CRC and is believed to be essential for the establishment of metastasis, therapy resistance, and disease recurrence [4,7,11,12].

LGR5 plays an important role in phenotypic plasticity in both intestinal tissue regeneration and cancer, regulating cellular behavior through the WNT signaling pathway (see below and [13]). Several lines of evidence also point to the Yes-associated pathway (YAP) as a common denominator in phenotypic stem cell plasticity, with both YAP and activator protein 1 (AP-1) driving oncofetal reprogramming [14]. Thus, stem cell plasticity may also mirror the situation during embryonic development, during which cells are highly adaptable.

The potential for LGR5+ stem-cell-like cells to both drive cancer growth and mediate treatment resistance has fueled increasing interest in targeting LGR5 therapeutically. However, the ability of LGR5+ stem-cell-like cells to undergo cellular reprogramming via phenotypic plasticity presents a significant challenge. Eliminating the LGR5+ cell population alone with LGR5-targeting drugs may not be sufficiently effective as monotherapy, because the LGR5+ cell population can readily be restored, through phenotypic plasticity, from the LGR5- cell population. Combining an additional therapeutic approach with an LGR5-targeted therapy, or by combining two different therapeutic approaches within one BsAb, may address the limitations of targeting LGR5 alone. An additional advantage of a BsAb is that a second binding arm directed to a tumor-specific antigen may facilitate preferential targeting of the LGR5+ stem-cell-like cell within a tumor rather than an LGR5+ ISC.

As a therapeutic class, BsAbs are becoming increasingly successful as medicines, with more than 15 clinically approved to date. The majority are directed against solid tumors, including bispecific T-cell engagers (CD3×DLL3 and CD3×EPCAM), a biparatopic BsAb targeting HER2, and two BsAbs targeting two receptors on a single cancer cell (EGFR×cMET and HER2×HER3), reviewed in [15].

This review focuses on the role of LGR5 in cancer biology, experiments leading to the selection of the EGFR×LGR5 BsAb petosemtamab as having the most potency, and its unique mechanisms of action. We also present key clinical results with petosemtamab in head and neck squamous cell carcinoma.

## 2. Biology of LGR5

**Figure 2:**
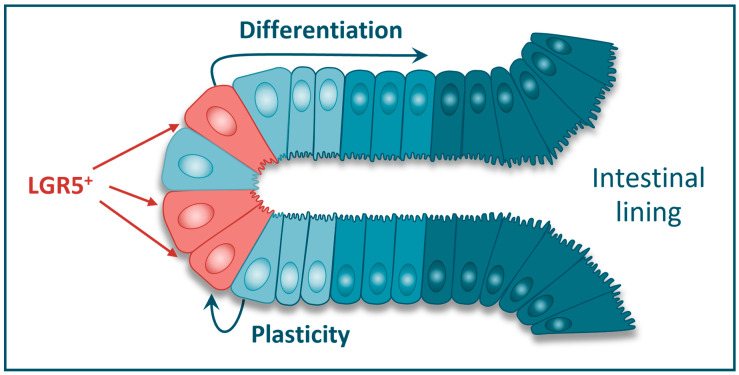
LGR5+ cells in the colonic crypt are believed to be the resident tissue stem cells of the normal colon. The LGR5+ cells provide a continuous supply of more differentiated intestinal epithelial cells for tissue regeneration and repair. LGR5+ cells can also arise through phenotypic plasticity from adjacent LGR5- cells.

LGR5 is expressed on the cell surface of a subset of stem cells in the gastrointestinal tract and elsewhere, including the liver, stomach, pancreas, kidney, breast, ovary, central nervous system, prostate, tongue, and other tissues (summarized in [16]). LGR5+ cells provide a continuous supply of more differentiated epithelial cells for tissue homeostasis, for example, in the colon where the epithelial lining is regenerated every 5 days (Figure 2) [16,17,18].

However, when the homeostatic balance is disrupted by severe injury and the consequent loss of the LGR5+ stem cell population, more differentiated LGR5- epithelial cells can give rise to LGR5+ stem cells, thereby facilitating tissue regeneration. This process of phenotypic cellular plasticity is well characterized in the intestine. Studies in murine models of intestinal damage have revealed that transient amplifying and terminally differentiated intestinal cells can restore a depleted intestinal stem cell niche [19,20]. Here, they adopt a transient Yes-associated pathway (YAP)-dependent fetal-like transcriptional program, highlighted by Ly6 (Sca-1) expression, which in turn promotes the expression of LGR5 and regeneration of the stem cell pool [21,22,23].

**Figure 3:**
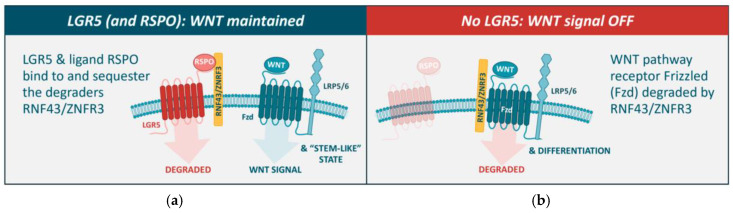
LGR5 potentiates the WNT–β-catenin signaling pathway that maintains the stem-cell-like phenotype, impeding cellular differentiation. (**a**) In the presence of LGR5 and RSPO, the E3 ubiquitin ligases RNF43/ZNFR3 are sequestered by LGR5, leading to the internalization and degradation of the LGR5 protein complex. In this process, RNF43/ZNFR3 are prevented from binding to and degrading the WNT receptor Fzd, and thus prevented from downregulating WNT signaling. (**b**) In the absence of LGR5 (or loss of WNT signaling), RNF43/ZNFR3 target Fzd for degradation, downregulating WNT signaling [18].

On a molecular level, LGR5 potentiates the WNT–β-catenin signaling pathway that maintains the cellular stem-cell-like phenotype, impeding cellular differentiation (Figure 3; reviewed in [18]). LGR5 (and the related homologs LGR4 and LGR6) is a high-affinity receptor for the R-spondin (RSPO) family of ligands. When bound to RSPO, the LGR5 protein associates with the E3 ubiquitin ligases RNF43/ZNRF3 [24], leading to internalization and degradation of the LGR5 protein complex [18,25]. In this process, RNF43/ZNRF3 is sequestered by the LGR5/RSPO complex and prevented from interacting with and degrading the WNT receptor Frizzled (Fzd). Consequently, Fzd expression and function and WNT–β-catenin pathway signaling are “on”, thereby conveying a more stem-cell-like cellular phenotype (Figure 3a). In contrast, when LGR5 is absent, the E3 ubiquitin ligases RNF43/ZNRF3 target Fzd for degradation (Figure 3b), downregulating WNT pathway signaling [18].

## 3. Role of LGR5 in Cancer

Multiple lines of evidence support a role for LGR5 in cancer. Human genetic studies have shown that mutations in WNT pathway genes, including LGR5, occur frequently in colorectal and other cancers [16,17,18] and are associated with worse clinical outcomes [26,27]. In specific experimental model systems of CRC, the LGR5+ subset of cells appears to be both necessary and sufficient to drive tumor establishment, growth, and metastasis [3,4,28].

**Figure 4:**
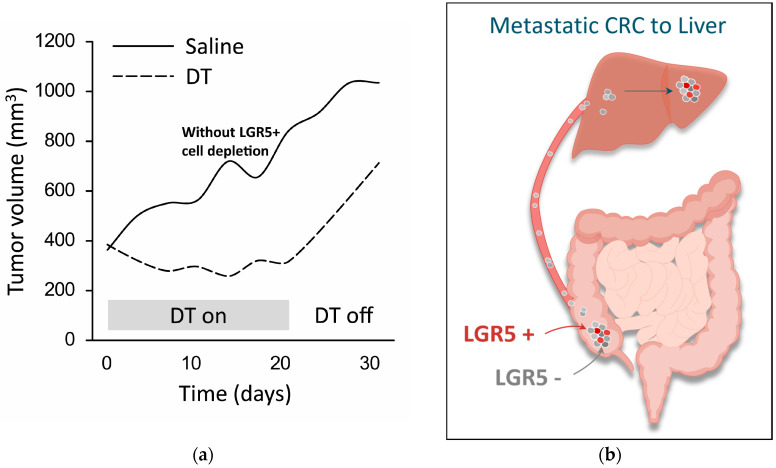
LGR5+ cells are essential for the growth of primary tumors and metastases. (**a**) Growth of genetically modified tumor model in mice is prevented during LGR5+ cell depletion (“DT on”, dashed line) but not in the absence of LGR5+ cell depletion (“DT off”, dashed line); tumor growth without cell depletion is shown for comparison (“Saline”, solid line) (adapted from [3]). This model employs mice carrying genetic mutations commonly found in human CRC, and also expressing a diphtheria toxin (DT) receptor fused to an enhanced green fluorescent protein under the genetic control of the LGR5 promoter (*Apc^min/+^*; *Kras^LSL-G12D/+^*; *Vil1^Cre^*; *Lgr5^DTR/eGFP^*). As a result, LGR5+ cells can be visualized and ablated selectively upon the administration of DT. (**b**) Liver micro-metastases are initially seeded by a population of LGR5- cells that have metastasized from a colorectal tumor in the cecum. The LGR5- cells within the micro-metastases subsequently undergo de-differentiation into LGR5+ stem-cell-like cells to facilitate further growth [32].

In preclinical models of human CRC, targeted depletion of the LGR5+ cells from a cancer does not fully eliminate the tumor mass, instead resulting in tumor stasis (Figure 4a) [3,4,7,29]. Tumor regrowth eventually occurs upon the reappearance of LGR5+ cells that derive from the LGR5- population, mirroring the transient fetal reprogramming and YAP-dependent phenotypic plasticity that occurs in normal (non-cancerous) tissue in response to injury [4,12,14,30]. A similar process appears to occur with metastases, where the initial seeding of micro-metastases seems to be mediated by LGR5- cells, which subsequently de-differentiate into LGR5+ stem-cell-like cells to facilitate further growth (Figure 4b) [4,31]. Consequently, more effective cancer therapy may be achieved by dual targeting of the LGR5- and LGR5+ cell populations to address both the LGR5- tumor bulk and initial metastatic seeding, and the LGR5+ cells that drive the growth of primary cancers and metastases.

Cancer therapy itself also appears to have an impact on the LGR5+ cell population. For example, chemotherapy leads to a residual population of quiescent, more stem-cell-like cells that can have lower LGR5 expression. Over time, LGR5+ cells can reappear and become a major source of long-term treatment failure [1,7,12,29,30,33,34,35].

Further, the inhibition of EGFR or other RTK signaling molecules can lead to the upregulation of LGR5 expression and treatment resistance [1,7,36,37,38,39]. One recent study has also identified upregulation of the transcription factor ASCL2, and enrichment of a population of proliferative stem-cell-like cells with high plasticity and inherent treatment resistance [39]. Conversely, the inhibition of LGR5 or elimination of the stem-cell-like cells can enhance sensitivity to targeted therapies [1,7,37,39]. Combining EGFR pathway inhibition with an LGR5-targeted approach more effectively treats cancer growth in a wide range of preclinical murine and human model systems [1,6,7,13,29,34,37]. Therefore, targeting the dynamically interacting oncogenic pathways of EGFR and LGR5 can result in a more effective treatment.

Despite our increased understanding of the role of the stem-cell-like subset of cells within solid tumors, monospecific targeting of these cells for therapeutic purposes has not, to date, been successful in the clinic. For example, the monoclonal anti-CD44v6 antibody bivatuzumab and the WNT-pathway-targeting porcupine inhibitor CGX1321 have been associated with limited monotherapy efficacy or unacceptable toxicity [40,41]. The rapid internalization of LGR5 in a ligand-independent fashion [25] also makes it an attractive potential target for antibody–drug conjugates (ADCs). At least three LGR5-targeting ADCs with different linkers, toxins, and drug-to-antibody ratios have been tested in preclinical models; however, no clinical trials on LGR5-directed ADCs have been reported to date [42,43,44,45,46].

A limitation of the use of monoclonal antibodies can be their relative inability to specifically target a cancer cell if the target antigen is widely expressed on other cell types, thereby giving rise to “off-tumor” binding to the intended target antigen where it is expressed on normal non-malignant cells. For example, anti-LGR5 antibodies may bind to LGR5 expressed on normal ISCs in addition to LGR5 expressed on the stem-cell-like cells within a tumor. One application of BsAbs is to ensure preferential binding to tumor cells as compared to normal cells, through the use of a second arm binding to an antigen expressed on the tumor cell. While petosemtamab can bind independently to cells expressing only the LGR5 antigen, the ability to simultaneously bind EGFR can in principle facilitate enhanced avidity-driven binding to dual-target-expressing cells within a tumor. And for petosemtamab specifically, one of the observed mechanisms of action requiring simultaneous binding to EGFR and LGR5 is the degradation of EGFR through LGR5-mediated internalization, described in further detail below.

## 4. Identification of Petosemtamab: An EGFR×LGR5 Bispecific Antibody

We used our Biclonics^®^ platform and leveraged the dual-targeting capabilities of a BsAb [47] to address both the survival and growth signals of these stem-cell-like cancer driver cells. A large biobank of patient-derived organoids (PDOs) was established from human CRC and adjacent normal colon tissue; these organoids are designed to re-create the cellular heterogeneity and organization of CRC or normal tissues [48,49]. We generated a diverse library of approximately 500 BsAbs targeting both HER family members (EGFR and HER3) and WNT pathway signaling receptors (LGR4, LGR5, ZNRF3, RNF43).

This approach involved the screening of synthetic phage antibody libraries as well as phage antibody libraries generated from immunized humanized transgenic common light chain mice (MeMo^®^), leading to the isolation of large panels of monoclonal antibodies specific for LGR4, LGR5, ZNRF3, RNF43, or EGFR [47]. Four EGFR binding arms, selected for their capacity to inhibit ligand-driven cell growth, and four previously identified HER3 binding arms, were combined with 10 LGR4, 17 LGR5, 18 ZNRF3, and 9 RNF43 binding arms into BsAbs. At the end of the selection and production process, each BsAb in the HER×WNT panel contained one arm directed at a WNT pathway target and the other arm directed at either EGFR or HER3.

**Figure 5:**
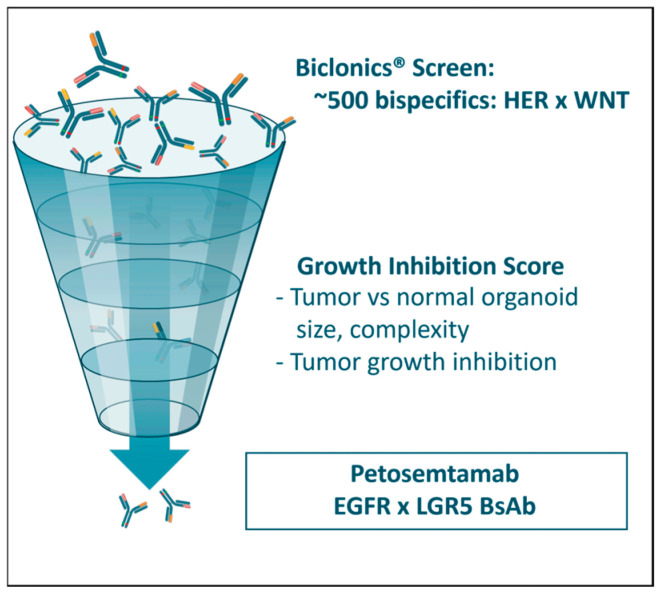
A large-scale screening assay was undertaken with approximately 500 Biclonics^®^ BsAbs to identify candidate molecules that preferentially inhibited the growth of cancer-derived, as compared to healthy-tissue-derived, organoids. Each BsAb contained one binding arm directed at a HER family member (HER3 or EGFR) and one binding arm directed at a WNT pathway protein (LGR4, LGR5, ZNRF3, or RNF43). The screen identified molecules targeting the combination of LGR5 and EGFR as the most promising for further study.

We evaluated the BsAb library by high-content imaging capturing the complexity of PDO responses to identify candidate molecules that demonstrated potent growth-inhibiting activity of cancer-derived, as compared to normal tissue-derived, organoids (Figure 5) [47]. This screening process led to the selection of 28 BsAbs that most robustly impacted the growth of human CRC-derived, as compared to normal tissue-derived, organoids: 14 were EGFR×WNT BsAbs and 14 were HER3×WNT BsAbs. Of these, the most promising BsAbs in terms of organoid growth inhibition activity were those that combined EGFR and LGR5. From this panel, we selected petosemtamab (also known as MCLA-158).

## 5. Petosemtamab Mechanisms of Action

**Figure 6:**
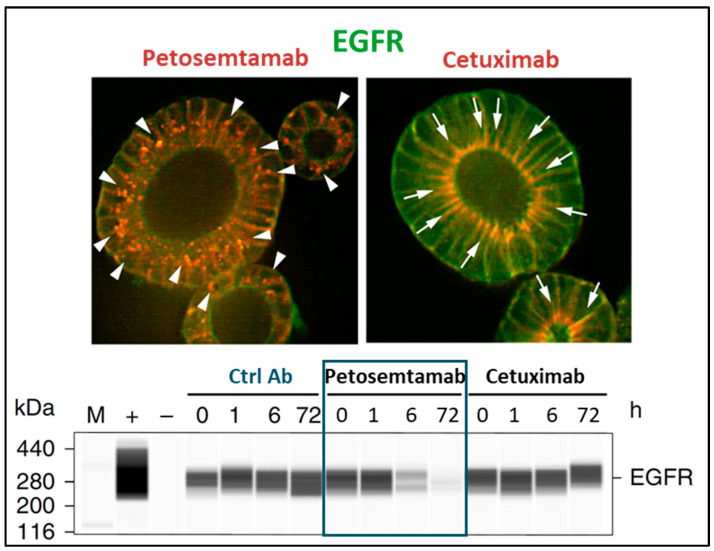
Petosemtamab mediates EGFR degradation in LGR5+ cells. Top: In CRC tumor-derived organoids (P18T), treatment with petosemtamab (red, arrowheads in **left** image) leads to internalization and disappearance of EGFR (green), as compared to treatment with cetuximab (red, arrows in **right** image) where EGFR staining (green) is maintained. **Bottom**: Western blot analysis demonstrating the disappearance of EGFR in P18T protein extracts upon treatment with petosemtamab, but not with cetuximab [47].

**Figure 7:**
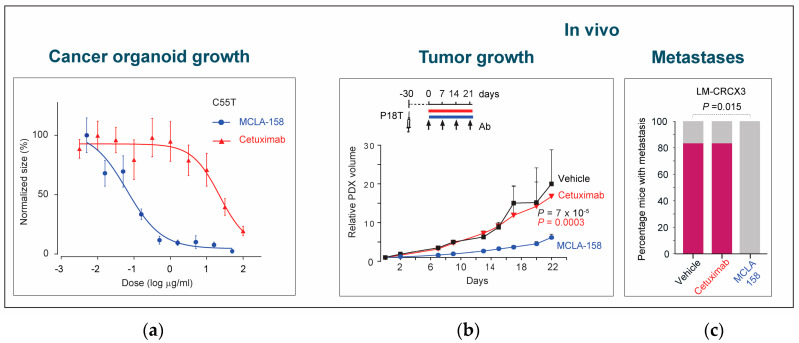
Petosemtamab (MCLA-158) effectively blocks tumor growth and metastases. (**a**) Petosemtamab provides more potent growth inhibition than cetuximab in a CRC organoid model in vitro. Shown are dose–response curves of PDO C55T (derived from a primary CRC tumor) cultured for 7–9 days in the presence of 2.5 ng/mL EGF, and increasing concentrations of petosemtamab or cetuximab. Each data point represents mean ± SEM of 6 independent cultures. (**b**) Petosemtamab provides more potent growth inhibition than cetuximab in a subcutaneous xenograft CRC model in vivo. Mice bearing subcutaneous P18T xenografts were treated with indicated antibodies (25 mg/kg) or PBS (vehicle) once per week. Tumor volumes relative to day 1 of treatment are shown. Each data point represents mean tumor volume ± SEM (29 tumors for cetuximab; 33 for petosemtamab; 29 for vehicle at day 0). (**c**) Petosemtamab is also more potent at preventing the development of liver metastases in an orthotopic xenograft CRC model in vivo. Mice bearing orthotopic CRC model LM-CRCX3 (derived from a lung metastasis) were treated with petosemtamab or cetuximab (0.05 mg per animal) once per week for 4 weeks, and the percentage of mice developing liver metastases was subsequently determined (indicated in pink; 6 per group). Petosemtamab completely prevented the development of metastases, whereas cetuximab exerted no effect. Adapted from [47].

The anticancer activity of petosemtamab may occur through multiple distinct mechanisms. Firstly, petosemtamab binds directly to a unique site on domain III of EGFR, preventing EGF ligand binding and EGFR-mediated cell signaling [47]. Secondly, petosemtamab binding to cells that express both LGR5 and EGFR facilitates the internalization and degradation of EGFR via the constitutively internalizing LGR5 protein (Figure 6) [18,47]. In contrast, no internalization or degradation of EGFR was observed with cetuximab, which binds only to EGFR, or with a monovalent EGFR-binding control BsAb lacking the LGR5 binding arm. Indeed, petosemtamab has been shown to be more effective than cetuximab in various preclinical models of cancer that express both LGR5 and EGFR, in terms of inhibiting organoid growth in vitro and inhibiting xenograft tumor growth or the outgrowth of metastases in vivo (Figure 7) [47]. Petosemtamab monotherapy has also shown striking antitumor activity clinically, greater than the historical efficacy of cetuximab, as described below.

Thirdly, petosemtamab exhibits Fc-mediated activation of the innate immune system by ADCP and enhanced ADCC, processes mediated by cells such as macrophages and natural killer (NK) cells. Petosemtamab is a fully human IgG1 antibody having low levels of fucose in the Fc region of the antibody. This lack of fucose increases the affinity of the Fc region of the antibody for certain immune-activating Fc receptors and maximizes ADCC activity. Enhanced ADCC activity has been shown to provide greater clinical benefit for other therapeutic antibodies such as obinutuzumab [50] and margetuximab [51] and may be important when petosemtamab is combined with immune checkpoint therapy such as pembrolizumab.

Finally, petosemtamab treatment itself, through the inhibition of EGFR, may lead to the upregulation of LGR5, a mechanism of resistance to EGFR inhibition [1,7,36,37,38]. In turn, increased LGR5 expression in the context of petosemtamab administration may lead to enhanced activity, through targeting of the emergent LGR5 antigen, serving as a further anchor for the above mechanisms of action, including ADCP and ADCC.

## 6. Promising Clinical Activity in HNSCC

Petosemtamab has demonstrated substantial clinical activity in patients with r/m HNSCC in initial, early interim results from clinical studies. The greater efficacy observed, as compared to historical studies of standard control therapies, may reflect a more effective targeting and elimination of the LGR5+ subset of cells within the cancer that may drive cancer growth and metastases.

In previously treated r/m HNSCC following one or more lines of treatment (2L+), petosemtamab monotherapy demonstrated a 36% confirmed overall response rate (ORR) in 75 evaluable patients, with median overall survival (mOS) of 11.5 months [52]. This efficacy compares favorably to historical estimates of standard treatments including cetuximab and single-agent chemotherapy, for which the ORR ranges between 6% and 19% and mOS ranges between 5.3 and 8.9 months [53,54,55,56]. Clinically, responses to petosemtamab monotherapy occur rapidly, with a median time to response occurring at the first radiographic assessment time point of approximately 2 months [52].

In the first-line (1L) treatment of r/m PD-L1+ HNSCC, petosemtamab added to standard pembrolizumab therapy demonstrated a 67% confirmed ORR in 24 evaluable patients, with robust responses across PD-L1 tumor levels and human papillomavirus status [57]. At the time of analysis, there was insufficient follow-up to estimate the median duration of response or survival. However, the early assessment of durability was promising with 20 of 24 patients remaining on study for longer than 4 months, and 18 patients continuing treatment. These results compare favorably to historical estimates of standard treatment with pembrolizumab alone in this setting, where the ORR ranges between 19% and 25% and median progression-free survival ranges between 2.8 and 3.2 months [58,59,60].

To date, the safety profile of petosemtamab has been generally favorable with low rates of grade 3 or greater (grade ≥ 3) adverse events overall and low rates of skin and gastrointestinal toxicity. The combination of pembrolizumab and petosemtamab showed no overlapping toxicities, with grade ≥ 3 treatment-emergent adverse events observed in 40% of patients. No specific type of grade ≥ 3 adverse events was observed in more than 10% of patients [56]. Infusion-related reactions (IRRs; grouped term) were manageable, with the most recent infusion administration regimen associated with IRRs in 45% of patients, and grade ≥ 3 IRRs observed in 9% of patients, virtually all occurring with the first treatment dose [52].

Based on these promising results, Merus N.V. is enrolling two phase 3 registrational trials for 1L r/m HNSCC (ClinicalTrials.gov Identifier: NCT06525220) and 2/3L r/m HNSCC (ClinicalTrials.gov Identifier: NCT06496178). Merus is also evaluating petosemtamab in metastatic CRC, both as a monotherapy and in combination with standard chemotherapy.

## 7. Conclusions

We have developed a novel therapeutic strategy to target epithelial cancers and, in particular, the subset of stem-cell-like cancer driver cells within these tumors. We selected petosemtamab, a novel and innovative EGFR×LGR5 Biclonics^®^ BsAb, from a screen of approximately 500 candidate antibodies tested for their preferential growth-inhibiting activity on cancer-derived, as compared to normal tissue-derived, organoids. Petosemtamab uniquely targets both LGR5+ stem-cell-like cells and the remaining LGR5- cells within a tumor through three distinct mechanisms: inhibition of EGF ligand binding and EGFR signaling, similar to other EGFR antibodies such as cetuximab; degradation of EGFR specifically in LGR5+ cells; and targeting LGR5+ and EGFR+ cells for immune-mediated destruction through ADCP and enhanced ADCC. At the same time, it also potentially blunts the oncogenic escape from EGFR inhibition through LGR5 upregulation. Early interim data from phase 2 clinical studies demonstrate encouraging clinical activity in r/m HNSCC that is substantially superior to historical results for standard approved therapies and may reflect a more effective treatment of the stem-cell-like LGR5+ cells within the cancer. We believe petosemtamab may also have broad potential in additional indications such as metastatic CRC, both as a monotherapy and in combination therapy, given the critical role of LGR5+ cells in CRC development. Petosemtamab may represent the first clinically effective therapy against the LGR5+ stem-cell-like cells within a tumor, frequently reported to be a central contributor to cancer growth, treatment resistance, and recurrence in CRC and other solid tumors.

## Abbreviations

The following abbreviations are used in this manuscript:

1L	First line
2L+	Second line or higher
ADC	Antibody–drug conjugate
ADCC	Antibody-dependent cellular cytotoxicity
ADCP	Antibody-dependent cellular phagocytosis
BsAb	Bispecific antibody
CRC	Colorectal cancer
EGF	Epidermal growth factor
EGFR	Epidermal growth factor receptor
FDA	Food and Drug Administration
Fzd	Frizzled
HER3	Human epidermal growth factor receptor 3
HNSCC	Head and neck squamous cell carcinoma
IgG1	Immunoglobulin G1
LGR5	Leucine-rich repeat-containing G-protein-coupled receptor 5
mOS	Median overall survival
ORR	Overall response rate
PD-L1	Programmed death-ligand 1
PDO	Patient-derived organoid
r/m	Recurrent/metastatic
RNF43	Ring finger protein 43
RSPO	R-spondin
RTK	Receptor tyrosine kinase
SEM	Standard error of the mean
WNT	Wingless-related integration site
ZNRF3	Zinc and ring finger 3
